# Temporal analysis of Z-Gradient coil eddy currents in tungsten collimator with different resistivities for SPECT/MRI

**DOI:** 10.1186/2197-7364-1-S1-A22

**Published:** 2014-07-29

**Authors:** Amine Samoudi, Karen Van Audenhaege, Günter Vermeeren, Micahel Poole, Luc Martens, Roel Van Holen, Wout Joseph

**Affiliations:** INTEC, Ghent University/iMinds, Ghent, Belgium; ELIS, Ghent University/iMinds, Ghent, Belgium; INM-4, Forschungszentrum Jülich GmbH, Jülich, Germany

Combining Single Photon Emission Computed Tomography (SPECT) with Magnetic Resonance Imaging (MRI) results in an interaction of the time-varying magnetic field gradients with the highly conducting tungsten collimator, which generates a secondary magnetic field causing spatial distortions in reconstructed MR images. Accurate simulations are important for the characterization of these eddy currents and to further optimize the gradient coils and the collimator design.

This study investigated the temporal variations of eddy currents in a tungsten collimator for pre-clinical SPECT/MRI due to only the z-gradient coil for different resistivities of tungsten, which can be obtained through additive manufacturing.

We modeled a z-gradient coil and a collimator using FEKO, a 3D electromagnetic simulation tool. A time analysis approach was used to generate the pulsed magnetic field gradient. The approach was validated with measurements using a 7T MRI scanner.

Simulations show that when tungsten with high resistivity (ρ=370 nΩ.m) is used, eddy currents generate an added magnetic field representing 1.72 % of the nominal gradient field in a Field Of View (FOV) of 3 cm. This percentage increases rapidly for tungsten with lower resistivities, which was expected since a higher resistivity implies lower current densities. A higher density of tungsten is preferred leading to low resistivity but results in stronger induced eddy currents. A compromise needs to be made between the eddy currents strength and the design of the collimator. Using the insights gained from these simulations, the next step is to optimize the design of the gradient coils and the collimator to further reduce the eddy current effects.

